# Interplay between miRNAs and host genes and their role in cancer

**DOI:** 10.1093/bfgp/elz002

**Published:** 2019-02-20

**Authors:** Baohong Liu, Yu Shyr, Jianping Cai, Qi Liu

**Affiliations:** 1 State Key Laboratory of Veterinary Etiological Biology, Key Laboratory of Veterinary Parasitology of Gansu Province, Lanzhou Veterinary Research Institute, Chinese Academy of Agricultural Sciences, Lanzhou, Gansu Province, China; 2 Center for Quantitative Sciences, Vanderbilt University Medical Center, Nashville, TN, USA; 3 Department of Biostatistics, Vanderbilt University Medical Center, Nashville, TN, USA; 4 Jiangsu Co-Innovation Center for Prevention and Control of Important Animal Infectious Diseases and Zoonoses, Yangzhou, China

**Keywords:** miRNA, intragenic miRNA biogenesis, host genes, interplay, cancer

## Abstract

MicroRNAs (miRNAs) are small endogenous non-coding functional RNAs that post-transcriptionally regulate gene expression. They play essential roles in nearly all biological processes including cell development and differentiation, DNA damage repair, cell death as well as intercellular communication. They are highly involved in cancer, acting as tumor suppressors and/or promoters to modulate cell proliferation, epithelial-mesenchymal transition and tumor invasion and metastasis. Recent studies have shown that more than half of miRNAs are located within protein-coding or non-coding genes. Intragenic miRNAs and their host genes either share the promoter or have independent transcription. Meanwhile, miRNAs work as partners or antagonists of their host genes by fine-tuning their target genes functionally associated with host genes. This review outlined the complicated relationship between intragenic miRNAs and host genes. Focusing on miRNAs known as oncogenes or tumor suppressors in specific cancer types, it studied co-expression relationships between these miRNAs and host genes in the cancer types using TCGA data sets, which validated previous findings and revealed common, tumor-specific and even subtype-specific patterns. These observations will help understand the function of intragenic miRNAs and further develop miRNA therapeutics in cancer.

## Introduction

MicroRNA (miRNA) is a class of small endogenous non-coding RNAs with a length between 20 and 24 nucleotides. They recognize and bind mRNA transcripts in a sequence-specific mode, resulting in degradation or translational repression of the corresponding mRNAs [1, 2]. The generation of miRNA from primary miRNA is a multi-step but fairly well understood process. After cleavage of primary transcripts to form precursor miRNAs in the nucleus, precursor miRNAs are exported into the cytoplasm for additional cleavage by an enzyme called Dicer to produce single-stranded mature miRNAs. Mature miRNAs are then loaded onto the RNA-induced silencing complex where they bind to 3′-UTR sequences of target genes. If the 3′-UTR of the target mRNA is fully complementary to the miRNA seed site, the mRNA is targeted for degradation. If it is partially complementary to the miRNA, the mRNA is targeted for translational inhibition [3–5]. In this way, an individual miRNA can post-transcriptionally regulate expression of hundreds of mRNAs, which profoundly alter gene expression and drive cells toward transformation.

Cancer is a complex disease involving multiple genes and pathways; therefore, it is not very useful to target one single gene for cancer treatment. A single miRNA can regulate expression of hundreds of genes involved in distinct cellular functions, which makes miRNAs the promising therapeutic targets. Since the miRNA dysregulation in cancer was first reported in 2002 [6], many studies have found miRNAs’ function in carcinogenesis including tumor suppressor miRNAs (ts-miRs) and oncogenic miRNAs (onco-miRs). The upregulated onco-miRs and downregulated ts-miRs lead to cancer initiation and progression [7, 8]. These miRNAs have attracted lots of attention as biomarkers and therapeutic targets. Controlling miRNAs transcriptional regulation by genome or RNA-editing tool has become a novel and promising approach [9, 10].

Although there is a general understanding of the generation of mature miRNA from primary miRNA, the transcriptional regulation of primary miRNAs is still unclear. The location of miRNAs is one major factor that determines how primary miRNAs are transcriptionally regulated. According to their genomic position, miRNAs can be classified into intragenic and intergenic miRNAs. Intragenic miRNAs, embedded within introns or exons of genes on the same strand, are believed to be co-regulated with their host genes by Pol II, whereas intergenic miRNAs, located between genes, are transcribed from their own Pol II or Pol III promoters. Recent studies, however, revealed that intragenic miRNAs, even intronic miRNAs, are not always co-transcribed with their host genes [11, 12]. Some intronic miRNAs have been found to interact with their host genes to affect their stability [13, 14]. More importantly, bioinformatics studies predicted that approximately 20% of intronic miRNAs target host mRNA transcripts in a feedback loop [13–15].

In this review, we outlined the complicated relationships between miRNAs and their host genes. Focusing on onco-miRs and ts-miRs in specific cancer types, we studied co-expression relationships between these miRNAs and host genes using TCGA data sets. We not only found common co-expression patterns, but also revealed tumor-specific and even subtype-specific relationships, which are very helpful for understanding transcriptional and post-transcriptional regulation of miRNAs and further developing miRNA therapeutics in cancer.

### Definition of intragenic miRNAs and their host genes

**Figure 1:**
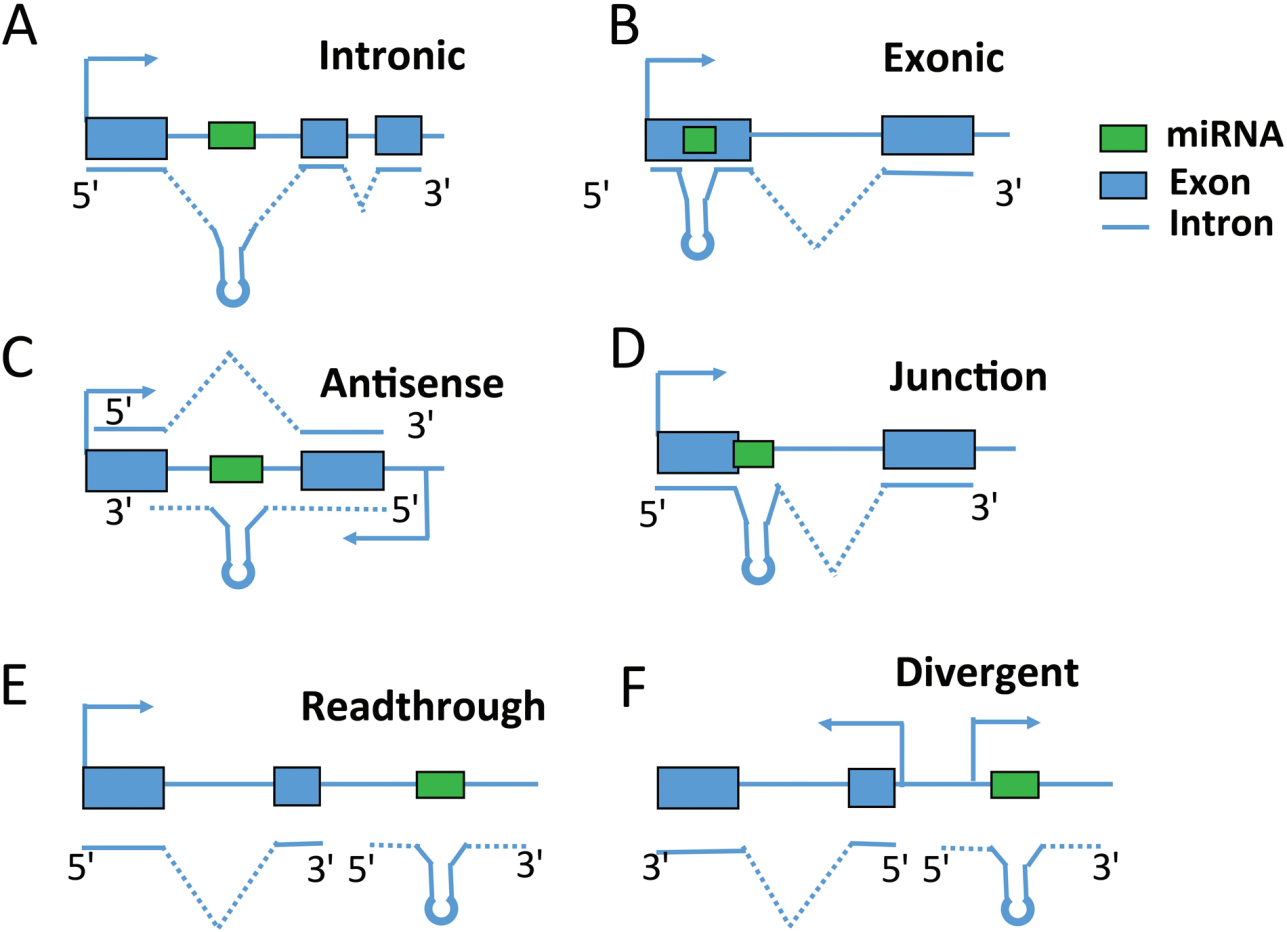
Intragenic miRNA classification. miRNA categories are defined based on their locations relative to exon, intron and gene regions.

Location of miRNAs is one of the hot topics in miRNA research since it is a major factor to determine how miRNAs are regulated. Based on their location, miRNAs can be classified into intragenic miRNAs and intergenic miRNAs. miRNAs located within protein-coding or non-coding genes are called intragenic miRNAs, while the genes in which the miRNAs are embedded are called host genes. Intragenic miRNAs can be further categorized into intronic miRNAs (miRNAs located in intronic regions), exonic miRNAs (miRNAs located in exonic regions), junction miRNAs (miRNAs located in gene intron–exon junctions) and antisense miRNAs (miRNAs located in the antisense strand of gene) (Figure 1). Intergenic miRNAs, located between genes, are believed to be transcribed from their own Pol II or Pol III promoters. However, recent studies revealed that there is a small percentage of intergenic miRNAs that are potentially co-transcribed with their neighboring genes through two mechanisms. One mechanism is called readthrough, where the transcription of one gene continues beyond the normal transcription termination site into intergenic regions. miRNAs located in the immediate (<4000 bp) downstream sense region of protein-coding or non-coding genes are likely to be transcribed by readthrough transcription [16] (Figure 1E). The other is called divergent transcription, defined as two polymerases transcribing on both sense and antisense directions from the same promoter. As a common feature for active promoters, divergent transcription generates long non-coding antisense transcripts coupled to active gene promoters [16–18] (Figure 1F). These two types of intergenic miRNAs (readthrough or divergent) can be classified into `intragenic’ category as well since they potentially share the same promoter with their neighboring genes, which are different from other intergenic miRNAs in transcriptional regulation. The neighboring genes that miRNAs are potentially co-transcribed with can also be called `host’ genes.

**Figure 2:**
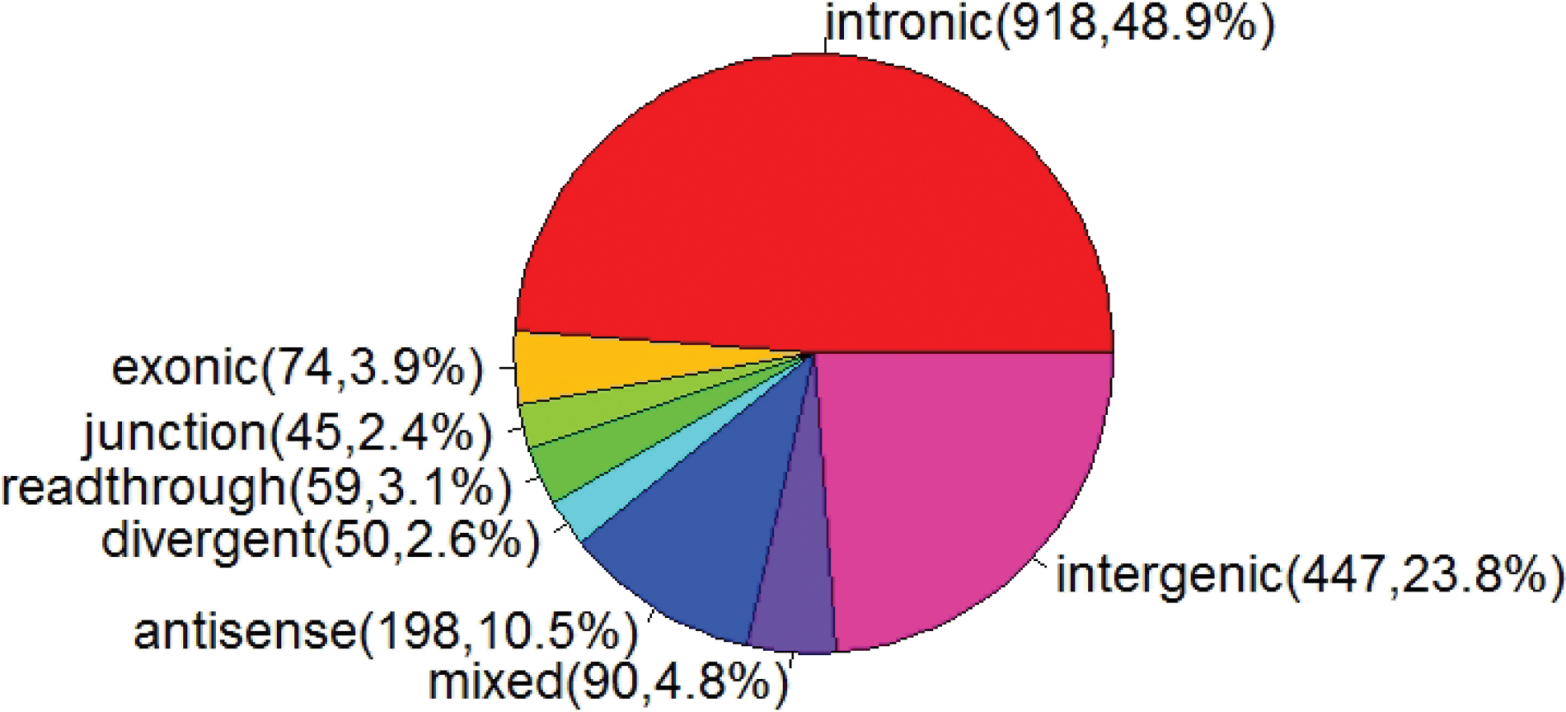
Pie chart of miRNA categories.

Previous studies have reported that more than half of miRNAs are intragenic miRNAs. To facilitate the analysis of genomic and structure features of intragenic miRNAs and host genes, miRIAD (http://www.miriad-database.org) provides a comprehensive knowledge on intragenic miRNAs by collecting public data from multiple sources, such as host gene function, miRNA and host gene expression and miRNA targets [19]. As gene annotation is improved and new miRNAs are identified, more intragenic miRNAs are classified [20]. To date, there are 1881 miRNAs in miRBase v21 [21–25]. Based on their relative location to protein-coding or non-coding genes (Figure 1), 1434 can be classified as intragenic miRNAs, among which 918 (48.8%), 74 (3.9%), 45 (2.4%), 59 (3.1%), 50 (2.6%), 198 (10.5%) are intronic, exonic, junction, readthrough, divergent and antisense miRNAs, respectively (Figure 2). There are 90 miRNAs (4.8%), which can not be assigned into one specific category. For example, an miRNA is located within the intron region of one transcript and also embedded in exon regions of another transcript. In this case, this miRNA is assigned into a `mixed’ category (Figure 2).

### Interplay between intragenic miRNAs and their host genes

Early evidence proposed a co-expression model suggesting that intronic miRNAs are derived from the same primary transcripts as their host genes. Intronic miRNAs use the transcriptional start sites (TSSs) of their host genes to initiate transcription. The primary transcript is then processed into mRNA and miRNAs by microprocessor and splicing. There is no interplay between microprocessor cleavage and splicing, resulting in the co-expression between miRNA and mRNA [26–28]. For example, the expression levels of miR-335 significantly correlated with *MEST* in 21 HCC cell lines, supporting the notion that the intronic miRNA is co-expressed with its host gene under the regulation of the host gene promoter [29]. Strongly correlated expression profiles between 175 miRNAs and their host genes across 24 different human organs have been reported [26]. An extreme case of coordination between intronic miRNA processing and splicing happens in very short introns, where the entire intron is so short that it is already a pre-miRNA without the involvement of microprocessor. These short introns, called mirtrons, are highly co-expressed with their host genes [30].

Increasing evidence, however, has shown that intronic miRNAs and host genes are not co-expressed as frequently as we previously expected due to two main reasons [31, 32]. One reason is that miRNAs have their own independent promoters. It has been found that over one-third of intronic miRNAs have their own promoters (Polymerase II or III), whose transcription is independent from their host genes [11, 33]. For example, it has been validated that miR-26b has an independent TSS, promoter signal and transcriptional factor from its host gene *CTDSP* [31]. Kim *et al*. [34] revealed that the splicing factor SRSF3 regulates the expression of miR-1908 independently from its host gene *FADS1*, suggesting that miR-1908 could be affected by its own transcription unit. Ozsolak *et al*. [35] reported that roughly 30% of intragenic miRNAs may be initiated independently especially if they are resided in large-sized (>5 kbp) intronic segments. The intronic regions are long enough to carry their own transcription units of pre-miRNA genes [32]. The other reason is the crosstalk between microprocessor cleavage and splicing [36]. In addition, the evolution conservation also contributes to the expression discordance between miRNAs and their hosts. He *et al*. [11] reported that evolutionary non-conserved intragenic miRNAs are less co-expressed with host genes than conserved ones. They suggest that most non-conserved miRNAs are expressed serendipitously and weakly but not necessarily always from the same transcripts as the host genes and some are co-expressed owing to shared chromatin dynamics. They then conjecture that for some of these their co-expression is beneficial and selection favors them to be what call ‘embedded’, meaning they evolve stronger co-expression, which in turn favors conservation of miRNA target sites on 3′-UTRs of desirable target genes [11].

The transcriptional regulation of exonic miRNAs is different from intronic miRNAs since Drosha processing of an exonic miRNA will inhibit production of the spliced host mRNA [37, 38]. Sundaram *et al*. [37] showed a clear inverse correlation between the expression pattern of miR-198 and *FSTL1* (host gene), which highlights the importance of this regulatory switch in controlling context-specific gene expression to orchestrate wound re-epithelialization.

The biogenesis of junction miRNAs is most complicated since there is competition between spliceosome and the microprocessor. When the splicing machinery does not recognize the internal exon, and the microprocessor components, rather than the spliceosome complex, bind to the RNA transcript at the pre-miRNA region to generate pre-miRNA. When the internal exon is recognized through interaction with specific splicing factors, the RNA secondary structure is released and splicing of the internal exon is carried out by the spliceosome before DGCR8 and Drosha bind to the pre-miRNA region. The spliced variant containing the alternative exon is the product and no pre-miRNA is made [39]. That is to say, junction miRNA is negatively regulated by splicing. Melamed *et al*. [39] studied an miRNA cluster including one exonic miRNA miR-410, one intronic miRNA miR-541 and one junction miRNA miR-412. They confirmed that the splicing and the processing of pre-miRNAs located in exon-intron junctions are mutually exclusive and alternative splicing is inversely correlated with levels of miR-412**.**

Readthrough and divergent miRNAs are special whose transcription is a byproduct of their neighboring genes. Readthrough miRNA is generated by a continuous transcription beyond a normal stop sign from an active gene (Figure 1E). For example, miR-21 is the most commonly over-expressed miRNA in cancer and a proven oncogene. miR-21 is located immediately downstream of the vacuole membrane protein-1 (*VMP1*) gene, which was reported to bypass the polyadenylation signals to include miR-21, thus providing a novel and independently regulated source of miR-21, termed VMP1–miR-21 [38]. Divergent miRNA is a product of bi-directional transcription of active promoters. In humans, some bidirectional promoter regions transcribing an miRNA and protein-coding gene simultaneously have been experimentally identified, e.g. the BTG4 promoter upstream of miR-34b and miR-34c [40], and the *POLR3D* promoter upstream of miR-320a on chromosome 8 [41].

In addition to the transcriptional regulation of intragenic miRNAs by their host genes, host genes are also subject to the regulation of miRNAs. The complicated and mutual regulation between miRNAs and host genes form a negative or positive feedback loop, where miRNAs play an antagonistic or synergetic role as a competitor or partner of a host gene [13, 14]. Bioinformatics studies have demonstrated that approximately 20% of intronic miRNAs were predicted to target their host genes [13]. One example is miR-338-3p, which inhibits the transcription of its host gene *AATK* by binding to its 3′-UTR [42]. Another example is miR-26b, which controls neuronal differentiation by repressing its host transcript, *ctdsp2* [43]. Such an interaction forms the `first-order’ negative-feedback circuit for fine-turning host gene expression. Besides direct regulation, an miRNA can negatively regulate its host gene function by targeting a gene whose product is a downstream effector of the host gene product. Such regulation could be termed `second-order’ negative feedback. For example, miR-641 is not predicted to target its host, *AKT2*, while its predicted targets, which include *PI3K, EGFR, PTEN, PDK1, RAS, MEK* and *ERK*, are in functional synergy with its host, giving credence to the second-order negative-feedback circuitry line of evidence [44].

**Table 1:** Summary of co-expression patterns between onco/ts-miRs and their host genes in specific cancer types

**miR cluster**	**miRNA**	**Host**	**Loc**	**Type**	**Cancer**	**Correlation in TCGA**	**Ref.**
miR-1	miR-1-1	MIR1-1HG	Intron	ts-miR	Thyroid; bladder cancer	–	[52, 53]
miR-9	miR-9-1	C1orf61	Intron	onco-miR	Breast cancer	0.39^**^	[54–56]
	miR-9-3	MIR9-3HG	Mix	ts-miR	Gastric cancer	0.25^**^	[56]
miR-15/16–1	miR-15a	DLEU2	Intron	ts-miR	CLL	–	[57, 58]
	miR-16-1					–	
miR-17-92	miR-17	C13ORF25	Mix	onco-miR	Lymphomas; lung cancer; renal cell cancer	–, 0.49^**^, 0.07	[57–59]
	miR-18a					–, 0.48^**^, 0.01	
	miR-19a					–, 0.50^**^, 0.11	
	miR-20a					–, 0.55^**^, 0.07	
	miR-19b1					–, 0.48^**^, 0.16	
	miR-92a1					–, 0.51^**^, 0.19	
miR-21	miR-21	VMP1	Read through	onco-miR	Breast cancer	0.52^**^	[60–65]
					Lung cancer	0.36^*^	
					Pancreas cancer	0.61^**^	
					Liver cancer	0.40	
					Gastric cancer	0.54^**^	
					Cervical cancer	0.39^**^	
					Hematopoietic cancer	–	
miR-23b	miR-23b	C9ORF3	Intron	ts-miR	Gastric cancer	0.65^**^	[66]
miR-26a	miR-26a-1	CTDSPL	Intron	ts-miR	Osteosarcoma; colorectal cancer	–,–	[67–69]
	miR-26a-2	CTDSP2				–,–	
miR-26b	miR-26b	CTDSP1	Intron	ts-miR	Colorectal cancer	–	[67, 69]
miR-27b	miR-27b	C9ORF3	Intron	ts-miR	Prostate cancer	0.62^**^	[60, 70]
miR-30c	miR-30c-1	NFYC	Intron	ts-miR	Prostate cancer	0.06	[71]
miR-33a	miR-33a	SREBF2	Intron	ts-miR	Prostate cancer	0.24^**^	[72]
miR-34b	miR-34b	BTG4	Divergent	ts-miR	Breast cancer	−0.05	[73]
					Prostate cancer	–	
miR-95	miR-95	ABLIM2	Intron	onco-miR	Liver cancer	0.75^**^	[74]
miR-101	miR-101-2	RCL1	Intron	ts-miR	Gastric cancer	0.17^*^	[75]
miR-106b/25	miR-106b	MCM7	Intron	onco-miR	Breast cancer	0.68^**^	[76]
	miR-93					0.57^**^	
	miR-25					0.66^**^	
miR-107	miR-107	PANK1	Intron	ts-miR	Pancreatic cancer	0.22^*^	[77]
miR-125b	miR-125b-1	MIR100HG	Intron	onco-miR	Lung cancer	0.69^**^	[63]
	miR-125b-2	MIR99AHG				0.77^**^	
miR-126	miR-126	EGFL7	Intron	ts-miR	Pancreatic cancer	0.56^**^	[78]
					Breast cancer	0.13^*^	
					Lung cancer	0.28	
miR-128	miR-128-1	R3HDM1	Intron	ts-miR	AML	–	[79–81]
					Esophageal cancer	–	
					Glioblastoma	–	
miR-137	miR-137	MIR137HG	Exon	ts-miR	Neuroblastoma	–	[82, 83]
miR-139	miR-139	PDE2A	Intron	ts-miR	Endometrial; colorectal cancer	–	[67, 84, 85]
miR-149	miR-149	GPC1	Intron	ts-miR	Breast cancer	0.37^**^	[86]
miR-152	miR-152	COPZ2	Intron	ts-miR	Prostate cancer	0.31^**^ 0.70^**^	[67]
					Endometrial cancer		
miR-153	miR-153-1	PTPRN	Intron	ts-miR	Ovarian cancer	–	[87]
					Glioblastoma	–	
					Gastric cancer	−0.007	
	miR-153-2	PTPRN2			Ovarian cancer	–	[87]
					Glioblastoma	–	
					Gastric cancer	0.45^**^	
miR-155	miR-155	MIR155HG	Exon	onco-miR	B-cell cancers	–	[88–91]
					Glioblastoma	0.68^**^	
miR-181a	miR-181a-1	MIR181A1HG	Intron	onco-miR	Cervical cancer	–	[92, 93]
					Breast cancer	–	
	miR-181a-2	MIR181A2HG			Cervical cancer	–	
					Breast cancer	–	
miR-185	miR-185	TANGO2	Intron	ts-miR	Colorectal cancer	–	[94]
					Lung cancer	0.49^**^	
					Breast cancer	0.36^**^	
miR-186	miR-186	ARANB2	Intron	ts-miR	Glioblastoma	0.4^**^	[95]
miR-198	miR-198	FSTL1	Exon	ts-miR	Breast cancer	−0.04	[96]
miR-210	miR-210	MIR210HG	Intron	onco-miR	Pancreatic cancer	–	[97]
miR-218	miR-218-1	SLIT2	Intron	ts-miR	Breast cancer	0.17^**^	[98]
					Gastric cancer	0.66^**^	
	miR-218-2	SLIT3			Breast cancer	0.33^**^	118
miR-224/452	miR-224	GABRE	Intron	onco-miR	Gastric cancer; lung cancer	0.69^**^, 0.83^**^ 0.75^**^, 0.80^**^	[99]
	miR-452			ts-miR	Prostate cancer	0.58^**^, 0.63^**^	
miR-326	miR-326	ARRB1	Intron	ts-miR	Lung cancer	0.41^**^	[67, 100]
miR-335	miR-335	MEST	Intron	onco-miR	Ovarian cancer	0.66^**^	[101, 102]
				ts-miR	Gastric cancer	0.79^**^	[103, 104]
					Liver cancer	0.88^**^	
					Breast cancer	0.78^**^	
miR-338	miR-338	AATK	Intron	ts-miR	Breast cancer	0.61^**^	[105]
miR-340	miR-340	RNF130	Intron	onco-miR	Gastric cancer	0.35^**^	[99]
				ts-miR	Breast cancer	0.28^**^	[106]
miR-342	miR-342	EVL	Intron	ts-miR	Colorectal cancer	–	[107]
miR-346	miR-346	GRID1	Intron	ts-miR	Liver cancer	0.10	[108]
miR-449a/b	miR-449a	CDC20B	Intron	ts-miR	Neuroblastoma	0.23^**^, 0.61^**^	[109]
	miR-449b				Prostate cancer	0.09, −0.25^**^	[110]
miR-486	miR-486	ANK1	Intron	ts-miR	Esophageal cancer	0.31^**^	[111]
miR-488	miR-488	ASTN1	Intron	ts-miR	Gastric cancer	−0.2^*^	[112]
miR-489	miR-489	CALCR	Intron	ts-miR	Esophageal cancer	0.02	[113]
miR-491	miR-491	FOCAD	Intron	ts-miR	Colorectal cancer	–	[114]
miR-504	miR-504	FGF13	Intron	onco-miR	HSCC	–	[115–117]
					Neuroblastoma	–	
				ts-miR	Glioblastoma	0.67^**^	
miR-634	miR-634	PRKCA	Intron	ts-miR	Breast cancer	–	[118]
miR-1908	miR-1908	FADS1	Intron	onco-miR	Glioblastoma	−0.12^*^	[57, 58]
					Ovarian cancer	0.01	

Cells with `–’ means correlation coefficients were not available since the expression of miRNA and/or its host gene were undetected in this cancer type.

^*^Represents the FDR ≤ 0.01.

^**^Represents the FDR ≤ 0.001.

### Association between intragenic onco/ts-miRNAs and their host genes in cancer

Disruption of miRNA expression is frequently observed in cancer. They can act as oncogenes and/or tumor suppressors. Study of a large collection of chronic lymphocytic leukemias (CLLs) showed knockdown or knock out of miR-15a and miR-16-1 in approximately 69% of CLLs. This is the first evidence that miRNAs might be involved in the pathogenesis of human cancer [6]. Frixa *et al*. [45] reported that miR-128-3p, upregulated in lung cancer, targets Drosha and Dicer, two key enzymes of miRNAs processing, leading to the widespread down-regulation of miRNA expression that promotes lung cancer cell migration [46]. The capability of targeting a number of genes with diverse functions makes miRNAs ideal candidates to regulate core carcinogenesis process [47–50].

Most onco-miRs or ts-miRs are intragenic miRNAs. Increasing evidences demonstrated links between intragenic miRNAs and host genes in cancer. They act in synergistic or antagonistic ways, which is achieved by miRNA-mediated fine-tuning target gene expression functionally related to host genes [51]. Due to the close functional connection between intragenic miRNAs and host genes, it is necessary and important to interpret the function of these onco-miRs and ts-miRs in the context of host genes. TCGA provides a rich source of multi-dimensional omics data, which gives a unique opportunity to study the expression of intragenic miRNAs and their host genes simultaneously across multiple cancer types. A positively correlated expression patterns would indicate a co-regulation and/or synergistic relationship between host and intragenic miRNAs, while anti-correlated or uncorrelated expression patterns would suggest an independent or antagonistic process.

Known onco-miRs and ts-miRs, specific cancer(s) where they are involved in, their cognate host genes and their expression correlation coefficients in specific cancer(s) are listed in Table 1. Most intragenic onco-miRs and ts-miRs are embedded within intron regions of genes. They exhibited a strong co-expression relationship with their cognate host genes, suggesting co-transcription and/or potential cooperation in regulating cancer development. The results derived from TCGA data sets further validated previous findings. For example, miR-335 harbored within an intron of a protein-coding gene *MEST*, was known to be co-regulated with *MEST* by promoter hypermethylation in breast cancer cells [119], HCC [29] and gastric cancer [120]. Consistently, the expression of miR-335 and *MEST* are highly correlated in TCGA HCC (R = 0.88; FDR = 9.87e-05), breast (R = 0.78; FDR = 0) and gastric cancer (R = 0.79; FDR = 0), further validating of the co-regulation relationship between miR-335 and *MEST* is needed. MiR-106b-25 cluster, composed of the highly conserved miR-106b, miR-93 and miR-25, is embedded within an intron 13 of *MCM7*. Petrocca *et al*. [121] reported that the *MCM7* and miR-106b-25 cooperate in exerting their oncogenic function through different complementary mechanisms. Consistently, miR-106b-25 and *MCM7* are highly co-expressed in TCGA breast cancer (R = 0.68, 0.57, 0.66; FDR = 0, 0, 0), further demonstrating that they are derived from the same primary transcript. Meanwhile, intronic miR-224/452 cluster showed significant co-expression with their host gene *GABRE* in prostate cancer (R = 0.58, 0.63; FDR = 0, 0) [122].

**Figure 3:**
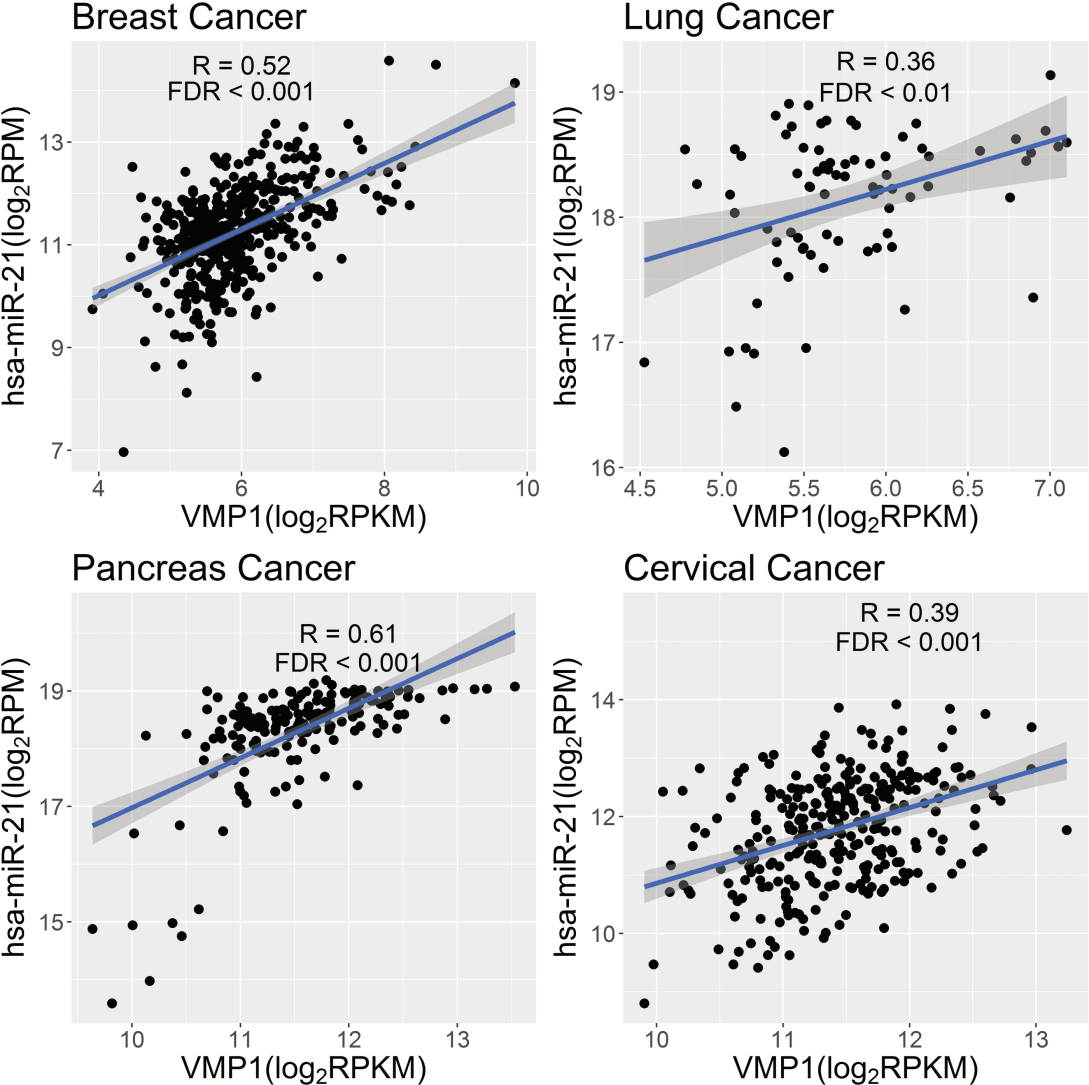
Scatter plot of expression abundance of miR-21 and its host gene VMP1 in multiple cancers.

**Figure 4:**
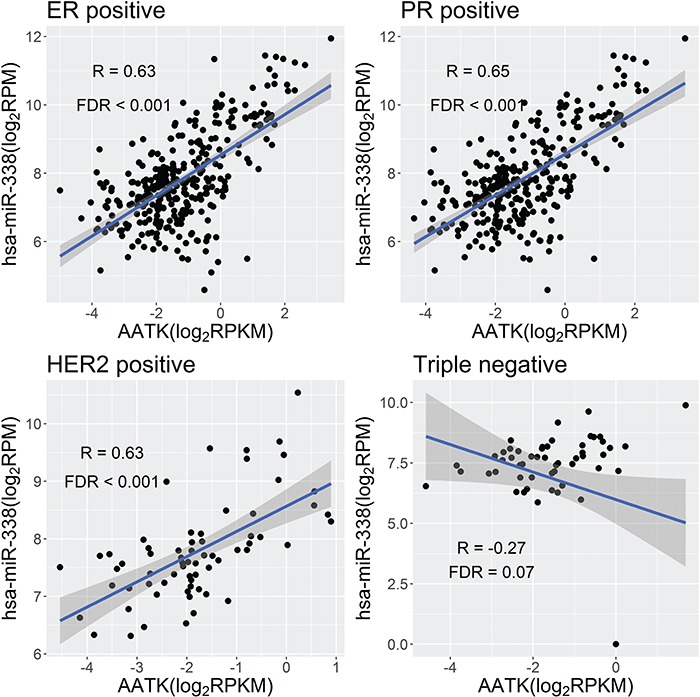
Scatter plot of expression abundance of miR-338 and its host gene AATK in ER/PR/HER2-positive and triple-negative breast cancers.

Although most intronic onco/ts-miRs are co-expressed with their host genes in cancers, there are some exceptions. For example, expression of miR-30c-1 is not correlated with its host gene *NFYC* (R = 0.06; FDR = 0.23) in prostate cancer, suggesting that it does not originate from the *NFYC* transcription unit. Several cryptic promoters and possible transcription factor binding sites have been detected in the intron regions of *NFYC* where miR-30c-1 is located, indicating miR-30c-1 is transcribed independent from *NFYC* [123]. Consistent with previous findings that miR-1908 is regulated by its own transcription unit rather than co-regulated with its host gene *FADS1* [34], we found that expressions of miR-1908 and *FADS1* are negatively correlated/uncorrelated in glioblastoma and ovarian cancer (R = −0.12, 0.01; FDR = 0.01, 0.87). In addition to known independent transcriptional relationships, TCGA data sets revealed novel uncorrelated/negatively correlated patterns between some intronic miRNAs and their host genes—for example, miR-346 and *GRID1* in liver cancer (R = 0.1; FDR = 0.81), miR-488 and *ASTN1* in gastric cancer (R = −0.2; FDR = 0.006) and miR-489 and *CALCR* in esophageal cancer (R = 0.02; FDR = 0.72). The uncorrelated/negative correlation indicated these miRNAs might have their own transcription mechanism rather than share the promoter as their host genes.

There are three onco/ts-miRs harbored within exonic regions, miR-137, miR-155 and miR-198. MiR-198 is located in the 3′-UTR region of protein-coding gene *FSTL1*, while the other two miRNAs are encoded in the exon of non-coding genes, *MIR137HG* and *MIR155HG*. MiR-198 is not co-expressed with its host gene *FSTL1* (R = −0.04 in breast cancer; FDR = 0.52), which validates previous finding about their mutually exclusive expression from a single transcript [37]. In contrast, miR-155 is co-transcribed with *MIR155HG* possibly because the gene itself is to generate the miRNA rather than produce other functional unit, leading to the high co-expression pattern in glioblastoma (R = 0.68; FDR = 0).

MiR-21, involved in many cancers, has been reported to be a readthrough miRNA from *VMP1*. The host gene, *VMP1*, was recently found to be involved in the process of tumor metastasis and it plays a vital role in balancing apoptosis and autophagy [38, 124]. miR-21 and *VMP1* are co-expressed across multiple cancer types (R = 0.52, 0.36, 0.61, 0.39; FDR = 0, 0.003, 0, 4.18e-12, in breast, lung, pancreas, and cervical cancers, respectively) (Figure 3). These findings further validated that miR-21 is the byproduct of transcription of *VMP1*.

MiR-34b and BTG4 are co-regulated by a bidirectional promoter [40]. It has been revealed that miR-34b has tumor suppressive ability in breast and prostate cancer [73]. However, TCGA data indicated that there is no correlation between expression of miR-34b and BTG4. Further research is needed to investigate whether this is caused by independent transcription or whether the co-transcription is disrupted by regulation feed-back between miRNAs and host genes.

Most interestingly, 15 out of 46 miRNA clusters show cancer-specific co-expression patterns with their host genes, suggesting the common involvement of cancer-specific post-transcription regulation—for example, miR-17-92 cluster, encoded the miR-17, miR-18a, miR-19a/b1, miR-20a and miR-92a1. miR-17-92 cluster, regarded as oncogene, was frequently overexpressed in lymphomas, lung cancer and renal cancer [57–59]. All miRNAs in the cluster showed strong correlation with the host gene C13orf25 in lung cancer (R = 0.49, 0.48, 0.58, 0.55, 0.48, 0.51; FDR = 2.26e-5, 2.89e-5, 1.35e-7, 1.08e-6, 2.82e-5, 9.90e-6), but weak/discordant correlation in renal cell cancer (R = 0.07, 0.01, 0.11, 0.07, 0.16, 0.19; FDR = 0.47, 0.93, 0.22, 0.47, 0.06, 0.02). These results suggest that miRNAs and host genes are co-transcribed from one transcript but renal cancer-specific post-transcription interferes the co-transcriptional regulation. In addition to cancer-specific pattern, some miRNAs even exhibit subtype-specific co-expression with their host genes in one cancer. For example, miR-338 is strongly co-expressed with its host gene AATK in ER/PR/HER2-positive breast cancers (R = 0.63, 0.65, 0.63; FDR = 0, 0, 2.49e-7), but this positive correlation is lost in triple-negative breast cancer (R = −0.27, FDR = 0.07) (Figure 4).

### Potential as the therapeutic targets in cancer

As master regulators of cellular processes, miRNAs have become promising therapeutic targets for cancer treatment. Upregulation of the expression of a ts-miR can induce apoptosis and senescence, block cell proliferation, self-renewal, invasion, metastasis, angiogenesis and drug resistance by selectively downregulating multiple genes [125]. Conversely, inhibiting the function or blocking the expression of an onco-miR in cancer cells can reactivate multiple tumor-suppressor genes, leading to tumor regression or even to tumor eradication [126].

Since most onco-miRs or ts-miRs are intragenic miRNAs, the observation that they are often co-expressed with host genes (Table 1), provides a new strategy to regulate miRNAs expression for cancer therapy. When miRNAs are derived from the transcription of host genes rather than have their own independent promoters, disruption of the expression of host genes or especially epigenetic modification of promoters of host genes is highly likely to change the miRNA expression. For example, miR-335, known to suppress breast cancer cell migration, is highly co-expressed with its host gene in breast cancer (Table 1). Furthermore, miR-335 expression is significantly negatively correlated with the MEST promoter DNA methylation level (R = −0.32; *P* = 0.0002), suggesting that promoter DNA demethylation would activate miR-335.

Since epigenetic modification is potentially reversible, it has been aggressively pursued as therapeutic strategy. Grady *et al*. [107] showed that there is synchronous epigenetic silencing of the intronic ts-miR miR-342 and its host gene *EVL* in colorectal cancer. Reconstitution of miR-342 activity induces apoptosis in colorectal cancer cells. Daniunaite *et al*. [127] analyzed the promoter methylation level of host genes of miR-155, miR-152, miR-137 and miR-31 in prostate cancer. They found that aberrant promoter methylation of the host genes is prostate cancer-specific. Downregulation of miR-155-5p significantly correlates with the promoter methylation level, which can be used as the promising diagnostic and/or prognostic biomarker of prostate cancer. The methylation status of host genes of particular miRNA as independent variables or in combinations with other signals might assist physicians in identifying prostate cancer patients with poor prognosis preoperatively. Besides, miR-152 and *COPZ2* were both silenced by hypermethylation in endometrial cancer. They are highly co-expressed in TCGA endometrial cancer data (R = 0.70; FDR = 0; Table 1). Tsuruta *et al*. [67] investigated the relationship between DNA hypermethylation and downregulation of miR-152 and *COPZ2* expression. They found that DNA hypermethylation-mediated silencing of miR-152 and *COPZ2* was a relatively frequent molecular event in endometrial cancer and inhibited cell growth in endometrial cancer cell lines, suggesting the epigenetic silencing of these genes to contribute to endometrial carcinogenesis. The restoration of miR-152 expression in endometrial cancer cell lines was sufficient to inhibit tumor cell growth in vitro and in vivo.

For the specific miRNA with dual identity that either onco-miR or ts-miR, there are different methylation pattern in different cancers. miR-224 and miR-452 are located in the intron 6 of the host gene γ-aminobutyric acid A receptor (*GABRE*). There are significant positive correlations between expressions of miR-224, miR-452 and *GABRE* in previous studies [122] and TCGA data sets across cancer types (R = 0.58~0.83; FDR = 0; Table 1), demonstrating that these three genes are transcriptionally co-regulated under the same promoter. Cui *et al*. [128] found that miR-224 was significantly upregulated and the CpG island in the promoter region of miR-224 was hypomethylated in lung cancer, suggesting the potent oncogenic role in lung cancer. In contrast, GABRE~miR-452~miR-224 locus is downregulated and hypermethylated in prostate cancer, suggesting tumor-suppressive role in prostate cancer [122]. Silencing/activating these miRNAs by epigenetic alterations of cognate host genes can be a potential way for cancer therapy.

Besides DNA methylation, histone acetylation is another way of epigenetic regulation. The ts-miRs of miR-15a/16–1, encoded within an intron region of the non-coding *DLEU2* gene, are downregulated in multiple tumor types while being frequently deleted in CLL. miR-15a/16–1 is believed to be transcribed from the *DLEU2* promoter. Kasar *et al*. [129] revealed that the transcription factor *BSAP* (B-cell-specific activator protein) directly interacts with *DLEU2*, resulting in repression of miR-15a/16–1 expression by negative regulation of the *DLEU2* promoter. Derepression of the *DLEU2* promoter via inhibition of histone deacetylation in combination with *BSAP* knockdown increased miR-15a/16–1 expression, leading to malignant B-cell death. Thus, therapy targeting enhanced host gene *DLEU2* transcription may augment CLL therapy. miR-126, harbored within the intron of the *EGFL7* gene, is downregulated in cancer cell lines and also in primary bladder and prostate tumors. miR-126 and one of the transcripts of *EGFL7* are concomitantly upregulated in cancer cell lines by inhibitors of DNA methylation and histone deacetylation. All these findings demonstrated that epigenetic changes can control the expression of onco/ts-miRs by directly controlling their host genes [130]. The knowledge of common and cancer-specific co-transcription patterns between miRNAs and host genes will help design therapeutic methods, targeting miRNA expression efficiently.

The advantage of miRNAs as therapeutic agents is based on their ability to concurrently target a large number of genes/pathways. However, this ability also causes a major concern on their toxicity due to potential off-target effects. While miRNAs target oncogenic pathways, they modulate other biological processes, which would cause far stronger side effects than generally appreciated [131]. Additionally, most existing strategies only focus on single miRNA or a family of miRNAs. Since miRNAs act cooperatively in tumor initiation and progression, modulating single miRNA expression has a limited effect. Tumor proliferation can be easily restored through bypassing the miRNA. Strategies to reprogram aberrant miRNA networks in cancer would be more effective [132]. Other challenges for developing miRNA therapeutics include low bioavailability, limited effective delivery and long-term safety [133].

## Conclusion

Dysregulation of miRNAs expression plays a critical role in human diseases, especially in cancer. There is a close functional connection and complicated interplay between miRNAs and host genes; therefore, it is essential to study the miRNA regulation in the context of their cognate host genes. Since most onco/ts-miRs are intragenic miRNAs, we studied the co-expression relationship between these miRNAs and host genes based on TCGA data sets. The common and cancer-specific co-expression relationships will facilitate the design of therapeutic methods to silence/activate onco/ts-miRs efficiently by epigenetic alterations of cognate host genes.

**Key Points**

- Although there is a general understanding of the generation of mature miRNA from primary miRNA, the transcriptional regulation of primary miRNAs is still unclear.
- Recent studies have shown that more than half of miRNAs are located within protein-coding or non-coding genes. Intragenic miRNAs and their host genes either share the promoter or have independent transcription.
- Most ts-miRs and onco-miRs are intragenic miRNAs; therefore, it is necessary and important to interpret their function in the context of host genes.
- The knowledge of common and tumor-specific co-expression patterns between miRNAs and host genes is very helpful for understanding transcriptional and post-transcriptional regulation of miRNAs and further developing miRNA therapeutics in cancer.

## Supplementary Material

point_to_point_response_1122_Supp_elz002

## Notes

**Baohong Liu** got her PhD in bioinformatics from Tongji University in 2011 and worked in State Key Laboratory of Veterinary Etiological Biology, Key Laboratory of Veterinary Parasitology of Gansu Province, Lanzhou Veterinary Research Institute, Chinese Academy of Agricultural Sciences from 2014. In 2017–18, she was a visiting scholar in Vanderbilt University.

**Yu Shyr** received his PhD in biostatistics from the University of Michigan in 1994. Now he is the Chair of Department of Biostatistics in Vanderbilt University.

**Jianping Cai** is the director of the laboratory of poultry research in State Key Laboratory of Veterinary Etiological Biology, Key Laboratory of Veterinary Parasitology of Gansu Province, Lanzhou Veterinary Research Institute, Chinese Academy of Agricultural Sciences. He is interested in the study of the genome, protein function for chicken parasites.

**Qi Liu** received her PhD at Shanghai Jiaotong University in 2003, completed her postdoc at Vanderbilt University and is now the assistant professor in Vanderbilt University.

## References

[1] Bartel DP MicroRNAs: target recognition and regulatory functions. Cell 2009;136(2):215–33. 19167326 10.1016/j.cell.2009.01.002 PMC3794896

[2] Nozawa M, Miura S, Nei M Origins and evolution of microRNA genes in plant species. Genome Biol Evol 2012;4(3):230–9. 22223755 10.1093/gbe/evs002 PMC3318440

[3] Ohtsuka M, Ling H, Doki Y, et al. MicroRNA processing and human cancer. J Clin Med 2015;4(8):1651–67. 26308063 10.3390/jcm4081651 PMC4555082

[4] Perron MP, Provost P Protein interactions and complexes in human microRNA biogenesis and function. Front Biosci 2008;13:2537–47. 17981733 10.2741/2865 PMC2901379

[5] Bartel DP MicroRNAs: genomics, biogenesis, mechanism, and function. Cell 2004;116(2):281–97. 14744438 10.1016/s0092-8674(04)00045-5

[6] Calin GA, Dumitru CD, Shimizu M, et al. Frequent deletions and down-regulation of micro-RNA genes miR15 and miR16 at 13q14 in chronic lymphocytic leukemia. Proc Natl Acad Sci U S A 2002;99(24):15524–9. 12434020 10.1073/pnas.242606799 PMC137750

[7] Oliveto S, Mancino M, Manfrini N, et al. Role of microRNAs in translation regulation and cancer. World J Biol Chem 2017;8(1):45–56. 28289518 10.4331/wjbc.v8.i1.45 PMC5329714

[8] Zhang M, Matyunina LV, Walker LD, et al. Evidence for the importance of post-transcriptional regulatory changes in ovarian cancer progression and the contribution of miRNAs. Sci Rep 2017;7(1):8171. 28811560 10.1038/s41598-017-08502-z PMC5557889

[9] Jing W, Zhang X, Sun W, et al. CRISPR/CAS9-mediated genome editing of miRNA-155 inhibits proinflammatory cytokine production by RAW264.7 cells. Biomed Res Int 2015;2015:1–7,326042. 10.1155/2015/326042 PMC4677169 26697483

[10] Huo W, Zhao G, Yin J, et al. Lentiviral CRISPR/Cas9 vector mediated miR-21 gene editing inhibits the epithelial to mesenchymal transition in ovarian cancer cells. J Cancer 2017;8(1):57–64. 28123598 10.7150/jca.16723 PMC5264040

[11] He C, Li Z, Chen P, et al. Young intragenic miRNAs are less coexpressed with host genes than old ones: implications of miRNA-host gene coevolution. Nucleic Acids Res 2012;40(9):4002–12. 22238379 10.1093/nar/gkr1312 PMC3351155

[12] Gao X, Qiao Y, Han D, et al. Enemy or partner: relationship between intronic micrornas and their host genes. IUBMB Life 2012;64(10):835–40. 22941954 10.1002/iub.1079

[13] Ma N, Wang X, Qiao Y, et al. Coexpression of an intronic microRNA and its host gene reveals a potential role for miR-483-5p as an IGF2 partner. Mol Cell Endocrinol 2011;333(1):96–101. 21146586 10.1016/j.mce.2010.11.027

[14] Li G, Wu X, Qian W, et al. CCAR1 5' UTR as a natural miRancer of miR-1254 overrides tamoxifen resistance. Cell Res 2016;26(6):655–73. 27002217 10.1038/cr.2016.32 PMC4897177

[15] Hinske LC, Galante PA, Kuo WP, et al. A potential role for intragenic miRNAs on their hosts' interactome. BMC Genomics 2010;11:533. 20920310 10.1186/1471-2164-11-533 PMC3091682

[16] Georgakilas G, Vlachos IS, Paraskevopoulou MD, et al. microTSS: accurate microRNA transcription start site identification reveals a significant number of divergent pri-miRNAs. Nat Commun 2014;5:5700. 25492647 10.1038/ncomms6700

[17] Seila AC, Calabrese JM, Levine SS, et al. Divergent transcription from active promoters. Science 2008;322(5909):1849–51. 19056940 10.1126/science.1162253 PMC2692996

[18] Seila AC, Core LJ, Lis JT, et al. Divergent transcription: a new feature of active promoters. Cell Cycle 2009;8(16):2557–64. 19597342 10.4161/cc.8.16.9305

[19] Hinske LC, Franca GS, Torres HA, et al. miRIAD-integrating microRNA inter- and intragenic data. Database (Oxford) 2014;2014:1–9. 10.1093/database/bau099 PMC4186326 25288656

[20] Lutter D, Marr C, Krumsiek J, et al. Intronic microRNAs support their host genes by mediating synergistic and antagonistic regulatory effects. BMC Genomics 2010;11:224. 20370903 10.1186/1471-2164-11-224 PMC2865499

[21] Kozomara A, Griffiths-Jones S miRBase: annotating high confidence microRNAs using deep sequencing data. Nucleic Acids Res 2014;42(Database issue):D68–73. 24275495 10.1093/nar/gkt1181 PMC3965103

[22] Kozomara A, Griffiths-Jones S miRBase: integrating microRNA annotation and deep-sequencing data. Nucleic Acids Res 2011;39(Database issue):D152–7. 21037258 10.1093/nar/gkq1027 PMC3013655

[23] Griffiths-Jones S miRBase: microRNA sequences and annotation. Curr Protoc Bioinformatics 2010;Chapter 12, Unit 12.9;1–10. 10.1002/0471250953.bi1209s29 20205188

[24] Griffiths-Jones S miRBase: the microRNA sequence database. Methods Mol Biol 2006;342:129–38. 16957372 10.1385/1-59745-123-1:129

[25] Griffiths-Jones S, Saini HK, Van Dongen S, et al., miRBase: tools for microRNA genomics. Nucleic Acids Res 2008;36(Database issue):D154–8. 17991681 10.1093/nar/gkm952 PMC2238936

[26] Baskerville S, Bartel DP Microarray profiling of microRNAs reveals frequent coexpression with neighboring miRNAs and host genes. RNA 2005;11(3):241–7. 15701730 10.1261/rna.7240905 PMC1370713

[27] Kim YK, Kim VN Processing of intronic microRNAs. EMBO J 2007;26(3):775–83. 17255951 10.1038/sj.emboj.7601512 PMC1794378

[28] Corcoran DL, Pandit KV, Gordon B, et al. Features of mammalian microRNA promoters emerge from polymerase II chromatin immunoprecipitation data. PLoS One 2009;4(4):1–10,e5279. 10.1371/journal.pone.0005279 PMC2668758 19390574

[29] Dohi O, Yasui K, Gen Y, et al. Epigenetic silencing of miR-335 and its host gene MEST in hepatocellular carcinoma. Int J Oncol 2013;42(2):411–8. 23229728 10.3892/ijo.2012.1724 PMC3583616

[30] Barron N, Sanchez N, Sanchez P, et al. MicroRNAs: tiny targets for engineering CHO cell phenotypes? Biotechnol Lett 2011;33(1):11–21. 20872159 10.1007/s10529-010-0415-5

[31] Sun Y, Ji F, Kumar MR, et al. Transcriptome integration analysis in hepatocellular carcinoma reveals discordant intronic miRNA-host gene pairs in expression. Int J Biol Sci 2017;13(11):1438–49. 29209147 10.7150/ijbs.20836 PMC5715526

[32] Li SC, Tang P, Lin WC Intronic microRNA: discovery and biological implications. DNA Cell Biol 2007;26(4):195–207. 17465886 10.1089/dna.2006.0558

[33] Schanen BC, Li X Transcriptional regulation of mammalian miRNA genes. Genomics 2011;97(1):1–6. 20977933 10.1016/j.ygeno.2010.10.005 PMC3019299

[34] Kim HR, Shin CH, Lee H, et al. MicroRNA-1908-5p contributes to the oncogenic function of the splicing factor SRSF3. Oncotarget 2017;8(5):8342–55. 28039456 10.18632/oncotarget.14184 PMC5352405

[35] Ozsolak F, Poling LL, Wang Z, et al. Chromatin structure analyses identify miRNA promoters. Genes Dev 2008;22(22): 3172–83. 19056895 10.1101/gad.1706508 PMC2593607

[36] Agranat-Tamir L, Shomron N, Sperling J, et al. Interplay between pre-mRNA splicing and microRNA biogenesis within the supraspliceosome. Nucleic Acids Res 2014;42(7):4640–51. 24464992 10.1093/nar/gkt1413 PMC3985634

[37] Sundaram GM, Common JE, Gopal FE, et al. ‘See-saw’ expression of microRNA-198 and FSTL1 from a single transcript in wound healing. Nature 2013;495(7439):103–6. 23395958 10.1038/nature11890

[38] Ribas J, Ni X, Castanares M, et al. A novel source for miR-21 expression through the alternative polyadenylation of VMP1 gene transcripts. Nucleic Acids Res 2012;40(14):6821–33. 22505577 10.1093/nar/gks308 PMC3413119

[39] Melamed Z, Levy A, Ashwal-Fluss R, et al. Alternative splicing regulates biogenesis of miRNAs located across exon-intron junctions. Mol Cell 2013;50(6):869–81. 23747012 10.1016/j.molcel.2013.05.007

[40] Toyota M, Suzuki H, Sasaki Y, et al. Epigenetic silencing of microRNA-34b/c and B-cell translocation gene 4 is associated with CpG island methylation in colorectal cancer. Cancer Res 2008;68(11):4123–32. 18519671 10.1158/0008-5472.CAN-08-0325

[41] Kim DH, Saetrom P, Snove O, et al. MicroRNA-directed transcriptional gene silencing in mammalian cells. Proc Natl Acad Sci U S A 2008;105(42):16230–5. 18852463 10.1073/pnas.0808830105 PMC2571020

[42] Kos A, Olde Loohuis F, Wieczorek ML, et al. A potential regulatory role for intronic microRNA-338-3p for its host gene encoding apoptosis-associated tyrosine kinase. PLoS One 2012;7(2):e31022. 10.1371/journal.pone.0031022 PMC3281898 22363537

[43] Dill H, Linder B, Fehr A, et al. Intronic miR-26b controls neuronal differentiation by repressing its host transcript, ctdsp2. Genes Dev 2012;26(1):25–30. 22215807 10.1101/gad.177774.111 PMC3258962

[44] Schmitt D, Tan M The enemy within: regulation of host genes by intronic microRNAs. Chemotherapy, 2014 3(e126):1–2.

[45] Frixa T, Sacconi A, Cioce M, et al. MicroRNA-128-3p-mediated depletion of Drosha promotes lung cancer cell migration. Carcinogenesis 2018;39(2):293–304. 29236960 10.1093/carcin/bgx134

[46] Bi L, Sun L, Jin Z, et al. MicroRNA-10a/b are regulators of myeloid differentiation and acute myeloid leukemia. Oncol Lett 2018;15(4):5611–9. 29552198 10.3892/ol.2018.8050 PMC5840650

[47] Zhang B, Pan X, Cobb GP, et al microRNAs as oncogenes and tumor suppressors. Dev Biol, 2007 302(1):1–12. 16989803 10.1016/j.ydbio.2006.08.028

[48] Palma Flores C, Garcia-Vazquez R, Gallardo Rincon D, et al. MicroRNAs driving invasion and metastasis in ovarian cancer: opportunities for translational medicine (Review). Int J Oncol 2017;50(5):1461–76. 28393213 10.3892/ijo.2017.3948

[49] Yan J, Ma C, Gao Y MicroRNA-30a-5p suppresses epithelial-mesenchymal transition by targeting profilin-2 in high invasive non-small cell lung cancer cell lines. Oncol Rep 2017;37(5):3146–54. 28405690 10.3892/or.2017.5566

[50] Cheng AM, Byrom MW, Shelton J, et al. Antisense inhibition of human miRNAs and indications for an involvement of miRNA in cell growth and apoptosis. Nucleic Acids Res 2005 33(4):1290–7. 15741182 10.1093/nar/gki200 PMC552951

[51] Hinske LC, Heyn J, Galante PA, et al. Setting up an intronic miRNA database. Methods Mol Biol 2013;936:69–76. 23007499 10.1007/978-1-62703-083-0_5

[52] Leone V, D'angelo D, Rubio I, et al MiR-1 is a tumor suppressor in thyroid carcinogenesis targeting CCND2, CXCR4, and SDF-1alpha. J Clin Endocrinol Metab 2011;96(9):E1388–98. 21752897 10.1210/jc.2011-0345

[53] Letelier P, Garcia P, Leal P, et al miR-1 and miR-145 act as tumor suppressor microRNAs in gallbladder cancer. Int J Clin Exp Pathol 2014;7(5):1849–67. 24966896 PMC4069933

[54] Chen D, Sun Y, Wei Y, et al. LIFR is a breast cancer metastasis suppressor upstream of the Hippo-YAP pathway and a prognostic marker. Nat Med 2012;18(10):1511–7. 23001183 10.1038/nm.2940 PMC3684419

[55] Ma L, Young J, Prabhala H, et al. miR-9, a MYC/MYCN-activated microRNA, regulates E-cadherin and cancer metastasis. Nat Cell Biol 2010;12(3):247–56. 20173740 10.1038/ncb2024 PMC2845545

[56] Meng Q, Xiang L, Fu J, et al. Transcriptome profiling reveals miR-9-3p as a novel tumor suppressor in gastric cancer. Oncotarget 2017;8(23):37321–31. 28418879 10.18632/oncotarget.16310 PMC5514911

[57] Macfarlane LA, Murphy PR MicroRNA: biogenesis, function and role in cancer. Curr Genomics 2010;11(7):537–61. 21532838 10.2174/138920210793175895 PMC3048316

[58] Kent OA, Mendell JT A small piece in the cancer puzzle: microRNAs as tumor suppressors and oncogenes. Oncogene 2006;25(46):6188–96. 17028598 10.1038/sj.onc.1209913

[59] Chow TF, Mankaruos M, Scorilas A, et al. The miR-17-92 cluster is over expressed in and has an oncogenic effect on renal cell carcinoma. J Urol 2010;183(2):743–51. 20022054 10.1016/j.juro.2009.09.086

[60] Meng F, Henson R, Wehbe-Janek H, et al. MicroRNA-21 regulates expression of the PTEN tumor suppressor gene in human hepatocellular cancer. Gastroenterology 2007;133(2):647–58. 17681183 10.1053/j.gastro.2007.05.022 PMC4285346

[61] Pan X, Wang ZX, Wang R MicroRNA-21: a novel therapeutic target in human cancer. Cancer Biol Ther 2010;10(12):1224–32. 21139417 10.4161/cbt.10.12.14252

[62] Zhu S, Si ML, Wu H, et al. MicroRNA-21 targets the tumor suppressor gene tropomyosin 1 (TPM1). J Biol Chem 2007;282(19):14328–36. 17363372 10.1074/jbc.M611393200

[63] Peralta-Zaragoza O, Deas J, Meneses-Acosta A, et al. Relevance of miR-21 in regulation of tumor suppressor gene PTEN in human cervical cancer cells. BMC Cancer 2016;16:215. 26975392 10.1186/s12885-016-2231-3 PMC4791868

[64] Zhang X, Gee H, Rose B, et al. Regulation of the tumour suppressor PDCD4 by miR-499 and miR-21 in oropharyngeal cancers. BMC Cancer 2016;16:86. 26867589 10.1186/s12885-016-2109-4 PMC4750294

[65] Reis ST, Pontes-Junior J, Antunes AA, et al. miR-21 may acts as an oncomir by targeting RECK, a matrix metalloproteinase regulator, in prostate cancer. BMC Urol 2012;12:14. 22642976 10.1186/1471-2490-12-14 PMC3431982

[66] Campos-Viguri GE, Jimenez-Wences H, Peralta-Zaragoza O, et al. miR-23b as a potential tumor suppressor and its regulation by DNA methylation in cervical cancer. Infect Agent Cancer 2015;10:42. 26622315 10.1186/s13027-015-0037-6 PMC4663735

[67] Tsuruta T, Kozaki K, Uesugi A, et al. miR-152 is a tumor suppressor microRNA that is silenced by DNA hypermethylation in endometrial cancer. Cancer Res 2011;71(20):6450–62. 21868754 10.1158/0008-5472.CAN-11-0364

[68] Tan X, Fan S, Wu W, et al. MicroRNA-26a inhibits osteosarcoma cell proliferation by targeting IGF-1. Bone Res 2015;3:15033. 27468358 10.1038/boneres.2015.33 PMC4948281

[69] Li Y, Sun Z, Liu B, et al. Tumor-suppressive miR-26a and miR-26b inhibit cell aggressiveness by regulating FUT4 in colorectal cancer. Cell Death Dis 2017;8(6):1–11,e2892. 28640257 10.1038/cddis.2017.281 PMC5520934

[70] Goto Y, Kojima S, Nishikawa R, et al. The microRNA-23b/27b/24-1 cluster is a disease progression marker and tumor suppressor in prostate cancer. Oncotarget 2014;5(17):7748–59. 25115396 10.18632/oncotarget.2294 PMC4202158

[71] Zhang J, Wang X, Wang Y, et al. Low expression of microRNA-30c promotes prostate cancer cells invasion involved in downregulation of KRAS protein. Oncol Lett 2017;14(1):363–8. 28693177 10.3892/ol.2017.6163 PMC5494817

[72] Karatas OF, Wang J, Shao L, et al miR-33a is a tumor suppressor microRNA that is decreased in prostate cancer. Oncotarget 2017;8(36):60243–56. 28947967 10.18632/oncotarget.19521 PMC5601135

[73] Majid S, Wang J, Shao L, et al. miRNA-34b inhibits prostate cancer through demethylation, active chromatin modifications, and AKT pathways. Clin Cancer Res 2013;19(1):73–84. 23147995 10.1158/1078-0432.CCR-12-2952 PMC3910324

[74] Ye J, Yao Y, Song Q, et al. Up-regulation of miR-95-3p in hepatocellular carcinoma promotes tumorigenesis by targeting p21 expression. Sci Rep 2016;6:1–12,34034. 27698442 10.1038/srep34034 PMC5048429

[75] Riquelme I, Tapia O, Leal P, et al. miR-101-2, miR-125b-2 and miR-451a act as potential tumor suppressors in gastric cancer through regulation of the PI3K/AKT/mTOR pathway. Cell Oncol (Dordr) 2016;39(1):23–33. 26458815 10.1007/s13402-015-0247-3 PMC4751587

[76] Liu Q, Wang J, Zhao Y, et al. Identification of active miRNA promoters from nuclear run-on RNA sequencing. Nucleic Acids Res 2017;45(13):e121. 28460090 10.1093/nar/gkx318 PMC5737662

[77] Imamura T, Komatsu S, Ichikawa D, et al. Depleted tumor suppressor miR-107 in plasma relates to tumor progression and is a novel therapeutic target in pancreatic cancer. Sci Rep 2017;7(1):5708. 28720759 10.1038/s41598-017-06137-8 PMC5515843

[78] Hamada S, Satoh K, Fujibuchi W, et al. MiR-126 acts as a tumor suppressor in pancreatic cancer cells via the regulation of ADAM9. Mol Cancer Res 2012;10(1):3–10. 22064652 10.1158/1541-7786.MCR-11-0272

[79] Mets E, Van Peer G, Van Deer Meulen J, et al. MicroRNA-128-3p is a novel oncomiR targeting PHF6 in T-cell acute lymphoblastic leukemia. Haematologica 2014;99(8):1326–33. 24895337 10.3324/haematol.2013.099515 PMC4116831

[80] Zhao L, Li R, Xu S, et al. Tumor suppressor miR-128-3p inhibits metastasis and epithelial-mesenchymal transition by targeting ZEB1 in esophageal squamous-cell cancer. Acta Biochim Biophys Sin (Shanghai) 2018;50(2):171–80. 29329360 10.1093/abbs/gmx132

[81] Papagiannakopoulos T, Friedmann-Morvinski D, Neveu P, et al. Pro-neural miR-128 is a glioma tumor suppressor that targets mitogenic kinases. Oncogene 2012;31(15):1884–95. 21874051 10.1038/onc.2011.380 PMC4160048

[82] Kozaki K, Imoto I, Mogi S, et al. Exploration of tumor-suppressive microRNAs silenced by DNA hypermethylation in oral cancer. Cancer Res 2008;68(7):2094–105. 18381414 10.1158/0008-5472.CAN-07-5194

[83] Althoff K, Beckers A, Odersky A, et al. MiR-137 functions as a tumor suppressor in neuroblastoma by downregulating KDM1A. Int J Cancer 2013;133(5):1064–73. 23400681 10.1002/ijc.28091

[84] Liu J, Li C, Jiang Y, et al. Tumor-suppressor role of miR-139-5p in endometrial cancer. Cancer Cell Int 2018;18:51. 29618950 10.1186/s12935-018-0545-8 PMC5879796

[85] Song M, Li C, Jiang Y, et al. MiR-139-5p inhibits migration and invasion of colorectal cancer by downregulating AMFR and NOTCH1. Protein Cell 2014;5(11):851–61. 25149074 10.1007/s13238-014-0093-5 PMC4225484

[86] Bischoff A, Huck B, Keller B, et al. miR149 functions as a tumor suppressor by controlling breast epithelial cell migration and invasion. Cancer Res 2014;74(18):5256–65. 25035394 10.1158/0008-5472.CAN-13-3319

[87] Zhou J, Xie M, Shi Y, et al. MicroRNA-153 functions as a tumor suppressor by targeting SET7 and ZEB2 in ovarian cancer cells. Oncol Rep 2015;34(1):111–20. 25954928 10.3892/or.2015.3952

[88] Yang L, Li C, Liang F, et al. MiRNA-155 promotes proliferation by targeting caudal-type homeobox 1 (CDX1) in glioma cells. Biomed Pharmacother 2017;95:1759–64. 28962081 10.1016/j.biopha.2017.08.088

[89] Due H, Svendsen P, Bodker JS, et al. miR-155 as a biomarker in B-cell malignancies. Biomed Res Int 2016;2016:1–14,9513037. 10.1155/2016/9513037 PMC4884835 27294145

[90] Tili E, Svendsen P, Bodker JS, et al. Modulation of miR-155 and miR-125b levels following lipopolysaccharide/TNF-alpha stimulation and their possible roles in regulating the response to endotoxin shock. J Immunol 2007;179(8):5082–9. 17911593 10.4049/jimmunol.179.8.5082

[91] O'Connell RM, Chaudhuri AA, Rao DS, et al. Inositol phosphatase SHIP1 is a primary target of miR-155. Proc Natl Acad Sci U S A 2009;106(17):7113–8. 19359473 10.1073/pnas.0902636106 PMC2678424

[92] Yue F, Cheng Y, Breschi A, et al. A comparative encyclopedia of DNA elements in the mouse genome. Nature 2014;515(7527):355–64. 25409824 10.1038/nature13992 PMC4266106

[93] Taylor MA, Sossey-Alaoui K, Thompson CL, et al. TGF-beta upregulates miR-181a expression to promote breast cancer metastasis. J Clin Invest 2013;123(1):150–63. 23241956 10.1172/JCI64946 PMC3533297

[94] Dong-Xu W, Jia L, Su-Juan Z MicroRNA-185 is a novel tumor suppressor by negatively modulating the Wnt/beta-catenin pathway in human colorectal cancer. Indian J Cancer 2015;52(Suppl 3):E182–5. 27453420 10.4103/0019-509X.186576

[95] Hui X, Jian J, Li Z, et al MiR-186 acts as a tumor suppressor by targeting TWIST1/2 and regulating the epithelial-mesenchymal transition in glioblastoma multiforme. Int J Clin Exp Pathol 2016;9(9):9706–14.

[96] Hu Y, Tang Z, Jiang B, et al. miR-198 functions as a tumor suppressor in breast cancer by targeting CUB domain-containing protein 1. Oncol Lett 2017;13(3):1753–60. 28454320 10.3892/ol.2017.5673 PMC5403721

[97] Ren D, Yang Q, Dai Y, et al. Oncogenic miR-210-3p promotes prostate cancer cell EMT and bone metastasis via NF-kappaB signaling pathway. Mol Cancer 2017;16(1):117. 28693582 10.1186/s12943-017-0688-6 PMC5504657

[98] Liu B, Tian Y, Li F, et al. Tumor-suppressing roles of miR-214 and miR-218 in breast cancer. Oncol Rep 2016;35(6):3178–84. 27109339 10.3892/or.2016.4749 PMC4869936

[99] Hashimoto Y, Akiyama Y, Yuasa Y Multiple-to-multiple relationships between microRNAs and target genes in gastric cancer. PLoS One 2013;8(5):1–11,e62589. 10.1371/journal.pone.0062589 PMC3648557 23667495

[100] Sun C, Huang C, Li S, et al miR-326 targets CCND1 and inhibits non-small cell lung cancer development. Oncotarget 2016;7(7):8341–59. 26840018 10.18632/oncotarget.7071 PMC4884997

[101] Scarola M, Schoeftner S, Schneider C, et al. miR-335 directly targets Rb1 (pRb/p105) in a proximal connection to p53-dependent stress response. Cancer Res 2010;70(17):6925–33. 20713524 10.1158/0008-5472.CAN-10-0141

[102] Cao J, Cai J, Huang D, et al. miR-335 represents an invasion suppressor gene in ovarian cancer by targeting Bcl-w. Oncol Rep 2013;30(2):701–6. 23708561 10.3892/or.2013.2482

[103] Sandoval-Borquez A, Polakovicova I, Carrasco-Veliz N, et al. MicroRNA-335-5p is a potential suppressor of metastasis and invasion in gastric cancer. Clin Epigenetics 2017;9:114. 29075357 10.1186/s13148-017-0413-8 PMC5645854

[104] Liu H, Li W, Chen C, et al. MiR-335 acts as a potential tumor suppressor miRNA via downregulating ROCK1 expression in hepatocellular carcinoma. Tumour Biol 2015;36(8):6313–9. 25804796 10.1007/s13277-015-3317-2

[105] Jin Y, Zhao M, Xie Q, et al. MicroRNA-338-3p functions as tumor suppressor in breast cancer by targeting SOX4. Int J Oncol 2015;47(4):1594–602. 26252944 10.3892/ijo.2015.3114

[106] Wu ZS, Wu Q, Wang CQ, et al. miR-340 inhibition of breast cancer cell migration and invasion through targeting of oncoprotein c-Met. Cancer 2011;117(13):2842–52. 21692045 10.1002/cncr.25860

[107] Grady WM, Parkin RK, Mitchell PS, et al. Epigenetic silencing of the intronic microRNA miR-342 and its host gene EVL in colorectal cancer. Oncogene 2008;27(27):3880–8. 18264139 10.1038/onc.2008.10

[108] Zhu W, Qing J, Ma L, et al. MiR-346 suppresses cell proliferation through SMYD3 dependent approach in hepatocellular carcinoma. Oncotarget 2017;8(39):65218–29. 29029425 10.18632/oncotarget.18060 PMC5630325

[109] Li F, Liang J, Bai L MicroRNA-449a functions as a tumor suppressor in pancreatic cancer by the epigenetic regulation of ATDC expression. Biomed Pharmacother 2018;103:782–9. 29684857 10.1016/j.biopha.2018.04.101

[110] Budd W Combinatorial analysis of tumorigenic microRNAs driving prostate cancer. PhD diss., Virginia Commonwealth University, 2012.

[111] Yi Y, Lu X, Chen J, et al. Downregulated miR-486-5p acts as a tumor suppressor in esophageal squamous cell carcinoma. Exp Ther Med 2016;12(5):3411–6. 27882172 10.3892/etm.2016.3783 PMC5103827

[112] Zhao Y, Lu G, Ke X, et al. miR-488 acts as a tumor suppressor gene in gastric cancer. Tumour Biol 2016;37(7):8691–8. 26738864 10.1007/s13277-015-4645-y

[113] Zhang B, Ji S, Ma F, et al miR-489 acts as a tumor suppressor in human gastric cancer by targeting PROX1. Am J Cancer Res 2016;6(9):2021–30. 27725907 PMC5043111

[114] Nakano H, Miyazawa T, Kinoshita K, et al. Functional screening identifies a microRNA, miR-491 that induces apoptosis by targeting Bcl-X(L) in colorectal cancer cells. Int J Cancer 2010;127(5):1072–80. 20039318 10.1002/ijc.25143

[115] Hu W, Chen CS, Wu R, et al. Negative regulation of tumor suppressor p53 by microRNA miR-504. Mol Cell 2010;38(5):689–99. 20542001 10.1016/j.molcel.2010.05.027 PMC2900922

[116] Kikkawa N, Kinoshita T, Nohata N, et al. microRNA-504 inhibits cancer cell proliferation via targeting CDK6 in hypopharyngeal squamous cell carcinoma. Int J Oncol 2014;44(6):2085–92. 24647829 10.3892/ijo.2014.2349

[117] Cui R, Guan Y, Sun C, et al. A tumor-suppressive microRNA, miR-504, inhibits cell proliferation and promotes apoptosis by targeting FOXP1 in human glioma. Cancer Lett 2016;374(1):1–11. 26854715 10.1016/j.canlet.2016.01.051

[118] Leivonen SK, Salhberg KK, Makela R, et al. High-throughput screens identify microRNAs essential for HER2 positive breast cancer cell growth. Mol Oncol 2014;8(1):93–104. 24148764 10.1016/j.molonc.2013.10.001 PMC5528509

[119] Png KJ, Yoshida M, Zhang XH, et al. MicroRNA-335 inhibits tumor reinitiation and is silenced through genetic and epigenetic mechanisms in human breast cancer. Genes Dev 2011;25(3):226–31. 21289068 10.1101/gad.1974211 PMC3034897

[120] Zhang JK, Li YS, Zhang CD, et al. Up-regulation of CRKL by microRNA-335 methylation is associated with poor prognosis in gastric cancer. Cancer Cell Int 2017;17:28. 28239297 10.1186/s12935-017-0387-9 PMC5314703

[121] Petrocca F, Vecchione A, Croce CM Emerging role of miR-106b-25/miR-17-92 clusters in the control of transforming growth factor beta signaling. Cancer Res 2008;68(20):8191–4. 18922889 10.1158/0008-5472.CAN-08-1768

[122] Kristensen H, Haldrup C, Strand S, et al. Hypermethylation of the GABRE~miR-452~miR-224 promoter in prostate cancer predicts biochemical recurrence after radical prostatectomy. Clin Cancer Res 2014;20(8):2169–81. 24737792 10.1158/1078-0432.CCR-13-2642

[123] Patel N, Tahara SM, Malik P, et al. Involvement of miR-30c and miR-301a in immediate induction of plasminogen activator inhibitor-1 by placental growth factor in human pulmonary endothelial cells. Biochem J 2011;434(3):473–82. 21175428 10.1042/BJ20101585 PMC3078570

[124] Guo XZ, Ye XL, Xiao WZ, et al. Downregulation of VMP1 confers aggressive properties to colorectal cancer. Oncol Rep 2015;34(5):2557–66. 26328607 10.3892/or.2015.4240

[125] Gambari R, Brognara E, Spandidos DA, et al. Targeting oncomiRNAs and mimicking tumor suppressor miRNAs: new trends in the development of miRNA therapeutic strategies in oncology (Review). Int J Oncol 2016;49(1):5–32. 27175518 10.3892/ijo.2016.3503 PMC4902075

[126] Nguyen DD, Chang S Development of novel therapeutic agents by inhibition of oncogenic microRNAs. Int J Mol Sci 2017;19(1):1–17. 10.3390/ijms19010065 PMC5796015 29280958

[127] Daniunaite K, Dubikaityte M, Gibas P, et al. Clinical significance of miRNA host gene promoter methylation in prostate cancer. Hum Mol Genet 2017;26(13):2451–61. 28398479 10.1093/hmg/ddx138

[128] Cui R, Meng W, Sun HL, et al. MicroRNA-224 promotes tumor progression in nonsmall cell lung cancer. Proc Natl Acad Sci U S A, 2015;112(31):E4288–97. 26187928 10.1073/pnas.1502068112 PMC4534291

[129] Kasar S, Underbayev C, Yuan Y, et al. Therapeutic implications of activation of the host gene (Dleu2) promoter for miR-15a/16-1 in chronic lymphocytic leukemia. Oncogene 2014;33(25):3307–15. 23995789 10.1038/onc.2013.291 PMC4508006

[130] Saito Y, Friedman JM, Chihara Y, et al. Epigenetic therapy upregulates the tumor suppressor microRNA-126 and its host gene EGFL7 in human cancer cells. Biochem Biophys Res Commun 2009;379(3):726–31. 19116145 10.1016/j.bbrc.2008.12.098

[131] Garzon R, Marcucci G, Croce CM Targeting microRNAs in cancer: rationale, strategies and challenges. Nat Rev Drug Discov 2010;9(10):775–89. 20885409 10.1038/nrd3179 PMC3904431

[132] Li X, Su Y, Sun B, et al. An artificially designed interfering lncRNA expressed by oncolytic adenovirus competitively consumes oncomiRs to exert antitumor efficacy in hepatocellular carcinoma. Mol Cancer Ther 2016;15(7):1436–51. 27196772 10.1158/1535-7163.MCT-16-0096

[133] Price C, Chen J MicroRNAs in cancer biology and therapy: current status and perspectives. Genes Dis 2014;1(1):53–63. 25473652 10.1016/j.gendis.2014.06.004 PMC4249815

