# Supplementary material for: Interplay between miRNAs and host genes and their role in cancer
Source: Brief Funct Genomics. 2019 Feb 20;18(4):255–66. doi: 10.1093/bfgp/elz002 (PMC6609535; doi:10.1093/bfgp/elz002)
Supplement: point_to_point_response_1122_Supp_elz002 [file point_to_point_response_1122_supp_elz002.docx]

**Interplay between miRNAs and host genes and their role in cancer (BFGP-18-0038)**

Reviewers' Comments to Author:

**Reviewer: 1**

Comments to the Author

**This paper provides a nice literature review on the relationship between intragenic miRNAs and host genes with a focus on its role in cancer, and performed interesting TCGA data analysis to validate these literature findings. Below are my comments.**

***Response:*** *We thank the reviewer very much for his/her insightful comments, which greatly improved the manuscript.*

1. **I have a few questions regarding Table 1. For each miR cluster, did the authors**

**investigate the co-expression patterns in TCGA for all cancer types or only for specific cancer types that have been reported in the literature?**

***Response:*** *Sorry for the confusion. In this review, we only focused on specific cancer types in which miRNAs have been reported to be oncogenes and/or tumor. For example, if one intragenic miRNA is known to be oncogenic in one cancer type, the co-expression between this miRNA and its host gene in this specific cancer type was investigated. We are working on another project where we explore the correlation pattern between all intragenic miRNAs and their host genes across all TCGA cancer types. This project will give a global view of common or cancer-specific regulation of intragenic miRNAs.*

*We clarify this in Abstract and also text on Page 11.*

*Abstract, “Focusing on miRNAs known as oncogenes or tumor suppressors in specific cancer types, it studied co-expression relationships between these miRNAs and host genes in the cancer types using TCGA datasets”*

*Page 11, “Known onco-miRs and ts-miRs, specific cancer(s) where they are involved in, their cognate host genes and their expression correlation coefficients in the specific cancer(s) are listed in table 1.”*

**Are the ones reported in Table 1 the statistically significant ones?**

***Response:*** *No, Table 1 includes the correlation coefficients between onco/ts-miRNAs and their host genes in specific cancer types even if the value is not statistically significant. Thanks to the reviewer’s comment, we realized that significance level should be provided as well, which would help conclude that a relationship really exists. In the revised Table 1, we labeled the correlation coefficients based on their significance level, where * indicates FDR<= 0.01 and ** indicates FDR<= 0.001.*

**What is the criterion for statistical significance?**

***Response:*** *Sorry for the unclearness. The original Table 1 reported the correlation coefficients regardless their significance level. In the revised Table 1, we used FDR<=0.01 as the criteria for statistical significance. We labeled those correlation values with “*” if FDR<=0.01 and “**” if FDR<=0.001.*

**Have multiple comparisons been adjusted?**

***Response:*** *Yes. The raw p values were adjusted by Benjamini–Hochberg procedure using all intragenic miRNAs****.***

**What does “-" represent?**

***Response:*** *Thanks for pointing this out!* ***“-“*** *indicates the correlation coefficient is unavailable due to the reason that expression of the miRNA and/or its host gene were undetected in the cancer type.*

*We added this information in Table 1.*

2. **The finding on cancer-specific co-expression pattern for miR-17-92 cluster is very**

**interesting. How general is this phenomenon? Does any other miR cluster show cancer-specific co-expression pattern?**

***Response:*** *Thanks for this insightful comment! We found this phenomenon is kind of common. Among 46 miRNA clusters we studied, 15 miRNA clusters showed cancer-specific coexpression patterns. Another example is miR-15/16-1, whose expression is strongly correlated with its host gene DLEU2 in Thymoma (R = 0.77,0.76) but uncorrelated in Uterine Corpus Endometrial Carcinoma (R = 0.07,0.09).*

*In addition, there is subtype-dependent co-expression between miRNAs and their host genes even in the same cancer type. In breast cancer, for example, the coexpression relationship between miRNAs and their host genes in Triple-negative breast cancers is very different from ER/PR/HER2-positive breast cancers. A specific example was shown in Figure 4. miR-338 is strongly coexpressed with its host gene AATK in ER/PR/HER2-positive breast cancers, but the correlation is weak and negative in Triple-negative breast cancers.*

*We added this information on Page 13.*

*“Most interestingly, 15 out of 46 miRNA clusters show a cancer-specific co-expression pattern with their host genes, suggesting the common involvement of cancer-specific post-transcription regulation. ”*

“*In addition to cancer-specific pattern, some miRNAs even exhibit subtype-specific coexpression with their host genes in one cancer. For example, miR-338 is strongly coexpressed with its host gene AATK in ER/PR/HER2-positive breast cancers (R = 0.63, 0.65, 0.63; FDR = 0, 0, 2.49e-7), but this positive correlation is lost in Triple-negative breast cancer (R = -0.27, FDR = 0.07) (Figure 4).”*

**3. The authors suggested epigenetic alterations as a promising approach to regulate the expression of onco/ts-miRs in cancer. Could they use TCGA data to demonstrate the correlation between miR expression and DNA methylation level of host gene?**

***Response:*** *Thanks for this great suggestion, which would further support the idea of epigenetic alteration as a promising approach to regulate the expression of onco/ts-miRs if the correlation between miR expression and DNA methylation level of host gene was demonstrated. miR-335, known to suppress breast cancer cell migration, is highly co-expressed with its host gene MEST in TCGA breast cancer (Table 1). Furthermore, miR-335 expression is significantly negatively correlated with the MEST promoter DNA methylation level (R=-0.32, p=0.0002), suggesting that promoter DNA demethylation would activate miR-335.*

*We added this example on Page 13 and 14.*

**Reviewer: 2**

Comments to the Author

**In this manuscript, Liu et al conducted a comprehensive review on the interplay between miRNAs and host genes and their role in cancer. This review is different from other previous reviews on miRNAs and cancer, as the focus of this review is in covering the host genes (i.e. genes where the miRNAs are embedded in the coding regions), and discussing more on their roles in driving various cancers. Some of the evidence are reviewed from published literatures, and the authors performed some comparisons using existing TCGA data sets. Overall, the review is well organized and the coverage of the topics is appropriate. The authors made extra efforts in correlating the gene expressions between miRNAs and their host genes using published data sets. However, this manuscript could be improved by clarifying the following points.**

***Response:*** *We greatly appreciate the reviewer’s valuable comments, which improved the work significantly.*

Major points:

**1. As pointed out by the authors, the expressions of these miRNAs are context-dependent, and in Table 1 and Figure 2, when the authors correlated the expressions of the miRNAs and their host genes, do the authors see stronger (or weaker) correlation by considering the subtypes? For example, in breast cancer, do the authors see a different correlation in ER/PR-positive, HER2-positive or Triple-negative breast cancer? In Lung cancer, do the authors see a different correlation in EGFR-mut and KRAS-mut subtypes?**

***Response:*** *This is a great question! We estimated the expression correlation between all intragenic miRNAs and their host genes in four breast cancer subtypes, ER/PR/HER2-positive, and Triple-negative breast cancers, respectively. We did find there are subtype-specific correlation patterns. Overall, Triple-negative breast cancers show a very different correlation pattern from other three subtypes (Figure 1 here). As one example, miR-338 is strongly coexpressed with its host gene AATK in ER/PR/HER2-positive breast cancers, but the correlation is weak and negative in Triple-negative breast cancers (Figure 4 in the manuscript).*

*We added this important finding on Page 13.*

*Page 13,“In addition to cancer-specific pattern, some miRNAs even exhibit subtype-specific coexpression with their host genes in one cancer. For example, miR-338 is strongly coexpressed with its host gene AATK in ER/PR/HER2-positive breast cancers (R = 0.63, 0.65, 0.63; FDR = 0, 0, 2.49e-7), but this positive correlation is lost in Triple-negative breast cancer (R = -0.27, FDR = 0.07) (Figure 4).”*

Figure 1. Heatmap of correlation coefficients between all intragenic miRNAs and their host genes in Triple-negative and ER/PR/HER2-positive breast cancers

2. **In Figure 2, are the values in RSEM or FPKM or TPM/CPM?**

***Response:*** *Sorry for the unclearness. We used log_2_RPM for miRNA expression and log_2_ RPKM for gene expression. We added the information in Figure 3.*

**Do the miRNAs correlated with outcomes in these cancers?**

***Response:*** *Yes. We focused on miRNAs that act as oncogene or tumor suppressors, most of which have been reported to be correlated with patient survival or tumor stage. For example, the high expression of miR-21 was reported to be associated with poor overall survival in multiple cancer types [1-4]. Unfortunately, we didn’t find the significant correlation between miR-21 expression and survival in TCGA data sets. However, Pan-Cancer miRNome Atlas reported eight cancer types where survival is significantly associated with miR-21 expression (*[*http://www.oncomir.org/oncomir/search_miR_surv.html*](http://www.oncomir.org/oncomir/search_miR_surv.html)*).*

# [1] Abue, M., et al., Circulating miR-483-3p and miR-21 is highly expressed in plasma of pancreatic cancer. Int J Oncol, 2015. 46(2): p. 539-47.

# [2] Zhu, W. and B. Xu, MicroRNA-21 identified as predictor of cancer outcome: a meta-analysis. PLoS One, 2014. 9(8): p. e103373

# [3] Yan LX, Huang XF, Shao Q, Huang MY, Deng L, et al. (2008) MicroRNA miR-21 overexpression in human breast cancer is associated with advanced clinical stage, lymph node metastasis and patient poor prognosis. RNA 14: 2348–2360

# [4] Yuan, Y., et al., Expressions of VEGF and miR-21 in tumor tissues of cervical cancer patients with HPV infection and their relationships with prognosis. Eur Rev Med Pharmacol Sci, 2018. 22(19): p. 6274-6279.

**3. In Table 1, currently, the table is a little bit hard to follow as every miRNA has correlation with different cancer types. This reviewer suggest that this could tidy up the table, maybe having different columns as the cancer types?**

***Response:*** *Thanks for pointing this out! In the revised Table 1, we used colors to denote cancer types. If one miRNA cluster acts as oncogene or tumor suppressor in multiple cancer types, colors were used to distinguish cancer types. Meanwhile, the correlation value in the cancer type was colored accordingly.*

4. **In table 1, what is the "-" means?**

***Response:*** *Thanks for pointing this out!* ***“-“*** *indicates the correlation coefficient is unavailable due to the reason that expression of miRNA and/or its host gene were undetected in the cancer type.*

*We added this information in Table 1.*

**5. How do the authors determine the correlation is significant, some correlation, with value of < 0.3 is displayed and annotated in Table 1, is it significant?**

***Response:*** *The table included all correlation values regardless of their significance level. Thanks to the reviewer’s comments, we realized that significance level should be provided, which would help conclude that a relationship really exists. In the revised Table 1, we labeled the correlation coefficients based on their significance level, where * indicates FDR<= 0.01 and ** indicates FDR<= 0.001.*

**6. The authors argued that miRNAs could be used as therapeutic targets, but delivering the miRNAs or mimetic could be challenging. Could you the authors discussed some of the pros and cons of targeting miRNAs in cancer?**

***Response:*** *Thanks for the great suggestion! We added some of the pros and cons of targeting miRNA in cancer on Page 17.*

*Page 17, “The advantage of miRNAs as therapeutic agents is based on their ability to concurrently target a large number of genes/pathways. However, this ability also causes a major concern on their toxicity due to potential off-target effects. While miRNAs target oncogenic pathways, they modulate other biological processes, which would cause far stronger side effects than generally appreciated [133]. Additionally, most existing strategies only focus on single miRNA or a family of miRNAs. Since miRNAs act cooperatively in tumor initiation and progression, modulating single miRNA expression has a limited effect. Tumor proliferation can be easily restored through bypassing the miRNA. Strategies to reprogram aberrant miRNA networks in cancer would be more effective [134]. Other challenges for developing miRNA therapeutics include low bioavailability, limited effective delivery, and long-term safety [135]. ”*

**Minor comments:**

1. **in the abstract, maybe change the "enemies" in the sentence "miRNAs work as partners or enemies of their host genes ...." to "competitors" or "antagonists"?**

***Response:*** *Thanks for the suggestion! We changed the “enemies” to “antagonists”.*
